# Strength of Gamma Rhythm Depends on Normalization

**DOI:** 10.1371/journal.pbio.1001477

**Published:** 2013-02-05

**Authors:** Supratim Ray, Amy M. Ni, John H. R. Maunsell

**Affiliations:** 1 Centre for Neuroscience, Indian Institute of Science, Bangalore, India; 2 Department of Neurobiology, Harvard Medical School, Boston, Massachusetts, United States of America; Albert Einstein College of Medicine, United States of America

## Abstract

Neuronal assemblies often exhibit stimulus-induced rhythmic activity in the gamma range (30–80 Hz), whose magnitude depends on the attentional load. This has led to the suggestion that gamma rhythms form dynamic communication channels across cortical areas processing the features of behaviorally relevant stimuli. Recently, attention has been linked to a normalization mechanism, in which the response of a neuron is suppressed (normalized) by the overall activity of a large pool of neighboring neurons. In this model, attention increases the excitatory drive received by the neuron, which in turn also increases the strength of normalization, thereby changing the balance of excitation and inhibition. Recent studies have shown that gamma power also depends on such excitatory–inhibitory interactions. Could modulation in gamma power during an attention task be a reflection of the changes in the underlying excitation–inhibition interactions? By manipulating the normalization strength independent of attentional load in macaque monkeys, we show that gamma power increases with increasing normalization, even when the attentional load is fixed. Further, manipulations of attention that increase normalization increase gamma power, even when they decrease the firing rate. Thus, gamma rhythms could be a reflection of changes in the relative strengths of excitation and normalization rather than playing a functional role in communication or control.

## Abstract 2

Manipulating a divisive normalization mechanism independently of attention in monkeys suggests that gamma power reflects excitation-inhibition interactions rather than plays a functional role in attentional processing.

## Author Summary

Brain signals often show a stimulus-induced rhythm in the “gamma” band (30–80 Hz) whose magnitude depends on attentional load, leading to suggestions that gamma rhythm plays a functional role in routing signals across cortical areas. However, gamma power also depends on simple stimulus features such as size or contrast, which suggests that gamma could arise from basic cortical processes involving excitation–inhibition interactions. One such process is divisive normalization, a mechanism that suppresses the response of a neuron by the overall activity of a large pool of neighboring neurons. Recent studies have shown that attention increases the strength of both excitation and normalization. We hypothesized that the increase in gamma power in an attention task is due to the effect of attention on excitation and normalization. By manipulating the normalization strength independent of attentional load in macaque monkeys, we show that gamma power increases with increasing normalization, even when attentional load is held fixed. Thus, gamma rhythms could be a reflection of changes in the relative strengths of excitation and normalization rather than playing a functional role in communication or control.

## Introduction

Modulations in gamma rhythms have consistently been observed during high-level cognitive processes such as attention [1]–[5], memory [6], feature-binding [7],[8], or conscious perception [9], leading to the suggestion that these rhythms play a functional role in high-level cognitive processing [7],[10]. However, several studies have shown that the magnitude and center frequency of the gamma rhythm depend on stimulus features such as contrast [11]–[13], orientation [14],[15], size [15],[16], and direction [12],[17], irrespective of the cognitive state, suggesting that gamma rhythms could be a reflection of basic cortical processes such as the interaction between excitation and inhibition [18]. Recent studies have suggested that selective attention, a high-level cognitive function often associated with gamma rhythms [1]–[5], is mediated through a sensory mechanism called normalization [19],[20]. Normalization is a form of gain control in which neuronal responses are reduced in proportion to the activity of a large pool of neighboring neurons [21],[22]. In the normalization model of attention, attention increases the excitatory drive to a neuron processing the attended stimulus. However, the increased excitatory drive also increases the strength of the normalization pool. The relative increase in the strength of normalization compared to excitation depends on several factors, such as the stimulus size and the focus of attention [20],[23], as well as tuning properties of the normalization pool [24], and these factors determine the overall effect of attention on the firing rate of the neuron.

The normalization model of attention, as well as other models (see Discussion), therefore predict that attention changes the relative strengths of excitation and inhibition. We hypothesized that the changes in gamma power observed with attention reflect the effect of attention on the underlying excitation and normalization strengths. In particular, we hypothesized that gamma power should increase with increasing normalization, even if attentional load is held fixed. We tested this hypothesis by recording single units and local field potentials (LFPs) from the middle temporal area (MT) of two macaque monkeys while they performed a task in which normalization and spatial attention were varied independently, and studying the effects of these manipulations on gamma power.

## Results

**Figure 1:**
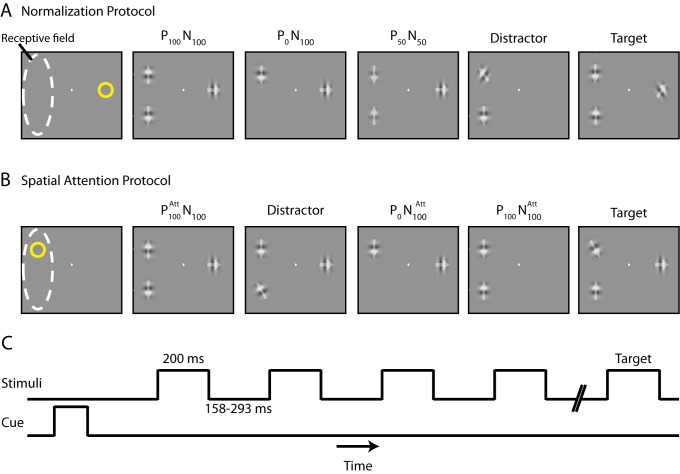
Experiment design. A series of drifting Gabor stimuli was flashed at each of three locations: two within the receptive field of the MT neuron being recorded and one outside the receptive field. The two stimuli inside the receptive field moved in the cell's preferred and null directions, the stimulus outside moved in the intermediate direction. The monkey was cued to attend to one of the three locations and was required to detect a change in the direction of the cued Gabor. (A) “Normalization Protocol”: The monkey attended outside the receptive field while the preferred and null stimuli were presented at 0%, 50%, or 100% contrasts, thus creating nine stimulus conditions. (B) “Spatial Attention Protocol”: The monkey attended to one of the locations inside the receptive field. (C) Time line of the protocols. See Materials and Methods for details.

To manipulate the strength of normalization, we cued the monkeys to attend to a stimulus outside the receptive field of an MT neuron while presenting two stimuli inside the receptive field—one moving in the cell's preferred direction and the second in the opposite (null) direction (“Normalization Protocol,” Figure 1A). The addition of a null stimulus, which by itself produces little excitation, decreases the response produced by the preferred stimulus alone, a phenomenon that has been explained using normalization [21],[22]. The addition of a null stimulus does not appreciably increase the excitatory drive received by the recorded neuron, but it increases the normalization strength considerably because other neurons in the normalization pool have different direction selectivities and therefore some neurons in the pool respond to the null stimulus also. Therefore, addition of a null stimulus increases normalization strength without any appreciable increase in excitation, and consequently decreases the firing rate. We manipulated normalization by varying the contrasts of the preferred and null stimuli inside the receptive field (each could take one of three contrasts: 0%, 50%, or 100%) while keeping the animal's attention directed away from the receptive field. We label each condition as P_x_N_y_, where x and y are the contrasts of the preferred and null stimuli. The stimuli were presented rapidly (200 ms) with a short interstimulus interval (158–293 ms; Figure 1C), which made it unlikely that the animals could adjust their attention in response to the variable contrast of stimuli within the duration of the presentations.

**Figure 2:**
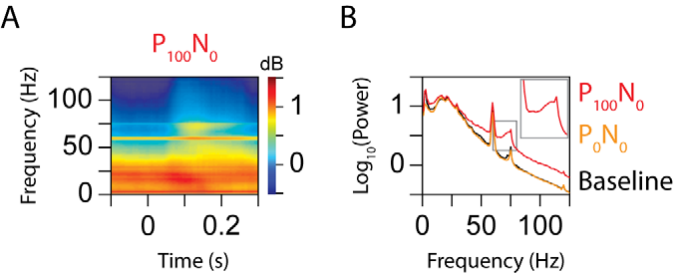
Time-frequency analysis. (A) Average time-frequency power spectrum of 96 sites for the P_100_N_0_ condition. The sharp horizontal lines at 60 and 75 Hz reflect the increase in power due to the line noise and monitor refresh rate, respectively. (B) Left panel shows the average power spectrum (as a function of frequency) during the P_100_N_0_ stimulus condition, computed by averaging the time-frequency power shown in Figure 2A between 50 and 250 ms (red trace). Spectrum for the P_0_N_0_ condition (orange) and the prestimulus baseline condition (black) are also shown for comparison. The inset shows the red trace at 2× magnification to highlight the narrow peak due to the monitor refresh rate.

Figure 2A shows the average time-frequency power (on a log scale) of 96 recording sites in the area MT of two monkeys (55 from Monkey 1 and 41 from Monkey 2; results were similar and individually significant for the two monkeys and hence the data were pooled) for the P_100_N_0_ condition (a single stimulus at 100% contrast moving in the preferred direction). Time-frequency analysis was done using the Matching Pursuit algorithm, which provided sufficient resolution to resolve any oscillatory activity related to normalization/attention as well as transient activity due to fast stimulus presentation rates (see Materials and Methods for details). Line noise and monitor refresh rate caused a sustained increase in power in the LFP, visible as two narrow horizontal lines at 60 and 75 Hz in Figure 2A. In addition, there was a prominent increase in power between 65 and 80 Hz starting around ∼100 ms after stimulus onset. Figure 2B shows the power spectrum (on a log scale) of the LFP, obtained by averaging the time-frequency power between 50 and 250 ms (red trace). For comparison, we also include the power spectrum when no stimulus was presented (P_0_N_0_ condition; orange trace) and the “baseline” spectrum obtained by averaging the power between 100 and 0 ms before stimulus onset for all nine normalization conditions (black trace). The baseline spectrum had slightly more power than the P_0_N_0_ spectrum (black curve is slightly above orange), which was expected because the baseline period contained some residual activity from the previous stimulus. The localized increase in gamma power between 65 and 80 Hz was reflected as a “bump” in the P_100_N_0_ spectrum, which was missing in both baseline and P_0_N_0_ spectra.

The gamma band increase observed between 65 and 80 Hz is not an artifact of the monitor refresh. Because the monitor refresh occurs at a fixed frequency, phase-locking of neurons to the monitor refresh rate is typically limited to a very narrow frequency band around the refresh rate, and in particular there is no evidence in the literature of such artifacts spreading to a broad frequency band. Further, even if the activity related to the monitor refresh rate varied with time (because the stimulus changed with time), it would cause an amplitude modulation of the 75 Hz sinusoid. The Fourier Transform of an amplitude modulated sinusoid is equal to the convolution of the Fourier Transform of the sinusoid (which produces a delta function at 75 Hz) and the Fourier Transform of the amplitude modulation. This is simply the Fourier Transform of the amplitude modulation centered at 75 Hz. Irrespective of the type of amplitude modulation introduced by the time-varying stimulus, the spread should be symmetric around 75 Hz, which was not the case. For the P_100_N_0_ condition, the artifact related to monitor refresh rate was visible as a narrow peak at 75 Hz that was distinct from the gamma band increase (the spectrum for the P_100_N_0_ condition around 75 Hz is enlarged in the inset). Further, gamma modulation was observed for the attention condition even when the stimulus conditions were identical (see below), which rules out the monitor refresh rate–related noise as the sole source of gamma power.

Although the use of Matching Pursuit resolved the line and monitor-related noise from ongoing oscillatory activity in the gamma band at high resolution, the results obtained using a traditional multitaper method [25],[26] were comparable and showed a prominent increase in power in the gamma range (Figure S1).

**Figure 3:**
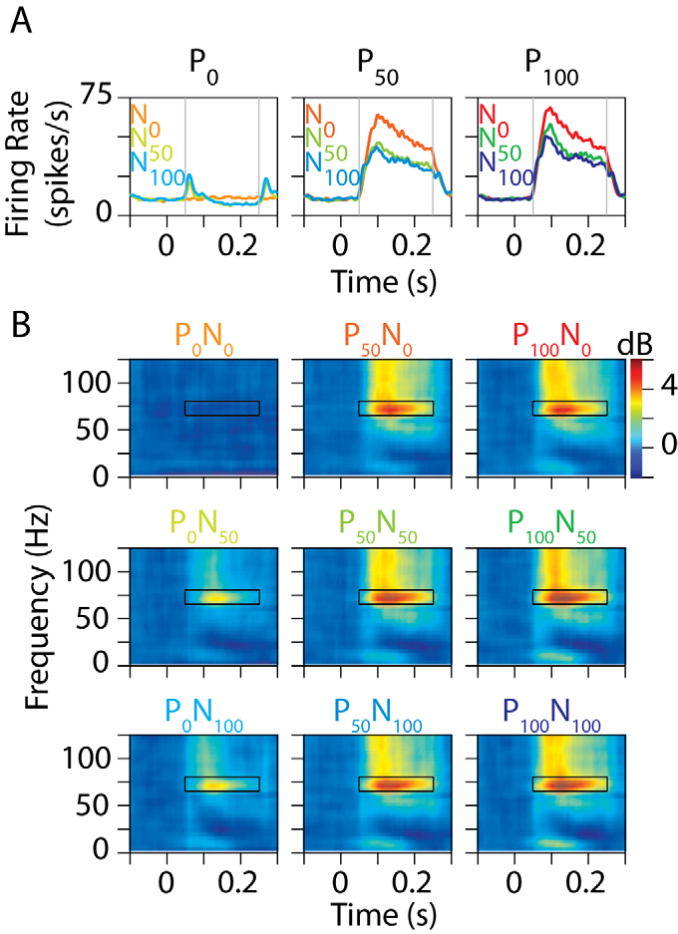
Gamma power depends on normalization. (A) Average firing rate of 96 MT neurons from two animals when two stimuli—one moving in the preferred direction and the other in the opposite (null) direction—were presented in the receptive field while the monkeys attended to a third stimulus outside the receptive field. The preferred and null stimuli were presented at 0%, 50%, or 100% contrast, yielding nine stimulus configurations. Each plot shows the data for a fixed value of the preferred contrast: 0% (i.e., no preferred stimulus; left panel), 50% (middle), or 100% (right). The different colored lines in each plot each represent a different null contrast: 0% (red; lower preferred contrasts have a lighter shade), 50% (green), or 100% (blue). The stimuli were presented for 200 ms. Firing rates were computed between 50 and 250 ms (gray lines). (B) Time-frequency power difference spectra, which represent the change in power relative to a prestimulus baseline (100 ms immediately before stimulus onset) for the nine stimulus conditions. Gamma rhythm was computed between 50 and 250 ms at 65 and 80 Hz, indicated by a black box in each plot.

**Figure 4:**
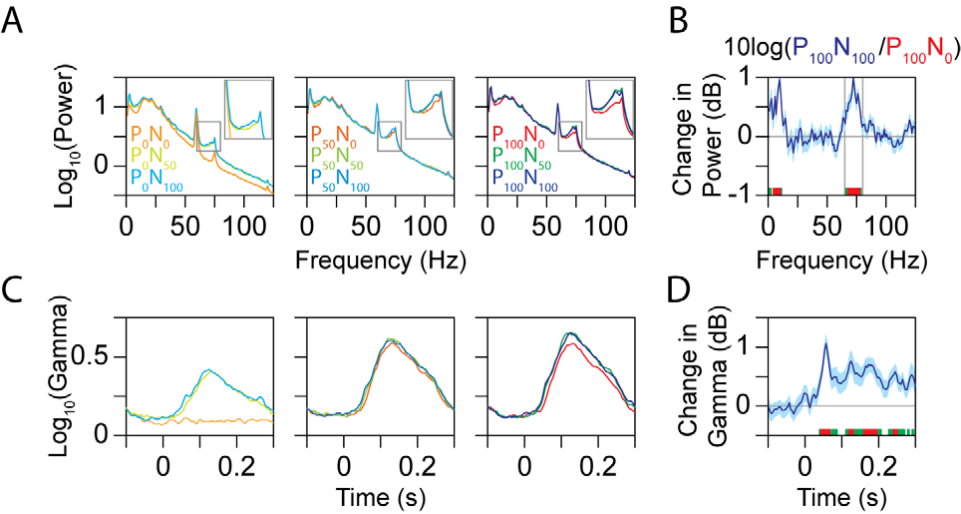
LFP power as a function of frequency and time. (A) Power spectra for different normalization conditions. (B) Change in power when both preferred and null stimuli are presented at 100% contrast (P_100_N_100_) versus when only a preferred stimulus is presented (P_100_N_0_). The shaded area represents the SEM. The frequency bins for which the change is significantly different are indicated by green (*p*<0.01, no Bonferroni correction) and red (*p*<0.05, Bonferroni corrected) squares at the bottom of the plot. Gray lines indicate the gamma range. (C) Gamma power (65–80 Hz, excluding 74–76 Hz) as a function of time, for different normalization conditions (same convention as A). (D) Change in gamma power between P_100_N_100_ versus P_100_N_0_ condition as a function of time (same convention as B).

Figure 3A shows the average firing rates when a stimulus moving in the neuron's preferred direction was presented at 0% (left), 50% (middle), and 100% (right) contrast, together with a null stimulus at 0% (red traces; lower preferred stimulus contrast is shown in a lighter shade), 50% (green), and 100% (blue) contrast. As expected from normalization, addition of a null stimulus decreased the firing rates. Figure 3B shows the change in LFP power relative to a common baseline period (Figure 2B, black trace) for different pairings of preferred (different columns) and null contrasts (different rows). Gamma rhythm was observed between 65 and 80 Hz, and its strength increased when a null stimulus was added (first versus second/third row). This increase was specific to the gamma band—for example, power did not increase in the high-gamma band (>80 Hz) with increasing normalization (Figure 3B, also see Figure 4B for comparison as a function of frequency).

To study these effects in more detail, we plotted the power between 50 and 250 ms as a function of frequency (Figure 4A) as well as the gamma power (between 65 and 80 Hz; excluding 74–76 Hz) as a function of time (Figure 4C), for all nine normalization conditions. Figure 4B and 4D show the change in power (in dB) between the P_100_N_100_ and P_100_N_0_ conditions as a function of frequency and time, respectively. In Figure 4B, the change was significant only in the gamma range and at very low frequencies (which was due to differences in transient activity; see Figure 3B). The change in gamma power started ∼50 ms after stimulus onset and persisted throughout the duration of the stimulus (Figure 4D).

**Figure 5:**
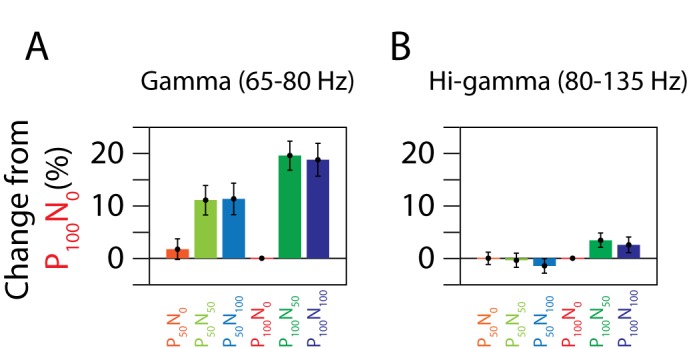
Percent changes in power from the P_100_N_0_ condition for the gamma (A) and hi-gamma (B) ranges.

To quantify the effect of normalization, we computed the total power in the gamma range (65–80 Hz, excluding 74–76 Hz; the analysis window is indicated by a black box in the panels of Figure 3B) and high-gamma range (80–135 Hz), for each normalization condition. Figure 5A shows the mean change in gamma power for different stimulus conditions relative to the P_100_N_0_ condition. Neurons in area MT typically have a low semi-saturation constant (σ in Text S1) and tend to saturate even for contrasts much less than 100% [27], so the results were similar for stimuli at 50% and 100% contrast (gamma power was not significantly different between P_50_N_0_ and P_100_N_0_ conditions; difference: 1.7%±2.0%, *p* = 0.39, *N* = 96, *t* test). However, gamma power increased significantly when a null stimulus at 50% or 100% contrast was added to a preferred stimulus at 50% or 100% contrast: relative changes in gamma power from P_100_N_0_ condition for P_50_N_50_, P_50_N_100_, P_100_N_50_, and P_100_N_100_ conditions were 11.1%±2.8%, 11.3%±3.0%, 19.6%±2.8%, and 18.8%±3.1%, respectively (*p* = 1.6×10^−4^, *p* = 2.9×10^−4^, *p* = 2.9×10^−10^ and *p* = 3.2×10^−8^, *N* = 96, *t* test). When analyzed separately for the two monkeys, the corresponding values were 10.5%±3.4%, 12.6%±4.3%, 25.6%±3.8%, and 27.3%±4.5% for Monkey 1 (*p* = 3.7×10^−3^, *p* = 4.6×10^−3^, *p* = 1.3×10^−8^, and *p* = 1.0×10^−7^, *N* = 55, *t* test) and 11.8%±4.7%, 9.5%±4.1%, 11.5%±3.7%, and 7.3%±3.5% for Monkey 2 (*p* = 0.02, *p* = 0.03, *p* = 0.003, and *p* = 0.04, *N* = 41, *t* test). On the other hand, the increases in high-gamma power (Figure 5B) for corresponding conditions were −0.4%±1.3%, −1.5%±1.4%, 3.4%±1.4%, and 2.5%±1.5%, respectively (*p* = 0.76, *p* = 0.3, *p* = 0.02, and *p* = 0.09, *N* = 96, *t* test). Thus, addition of a second stimulus inside the receptive field of a neuron, which increased normalization, increased the magnitude of the gamma rhythm even when attention was fixed outside the receptive field. However, increasing normalization had negligible effect at high-gamma frequencies.

Similar results were obtained using the multitaper method. Relative changes in gamma power (sum of power at 65, 70, and 80 Hz) from P_100_N_0_ condition for P_50_N_50_, P_50_N_100_, P_100_N_50_, and P_100_N_100_ conditions were 5.3%±2.4%, 8.0%±3.0%, 13.0%±2.7%, and 16.2%±3.4%, respectively (*p* = 0.03, *p* = 0.009, *p* = 5.2×10^−6^ and *p* = 7.3×10^−6^, *N* = 96, *t* test). For high-gamma power, the corresponding values were −0.2%±1.4%, −2.4%±1.3%, 2.2%±1.5%, and 1.0%±1.5%, respectively (*p* = 0.87, *p* = 0.07, *p* = 0.14, and *p* = 0.50, *N* = 96, *t* test).

Interestingly, while normalization is generally thought to be largely un-tuned for orientation [21],[22], the gamma rhythm was much stronger when a preferred stimulus was presented instead of a null stimulus (compare P_0_N_100_ versus P_100_N_0_ in Figures 3B; both should involve the same normalization signal). This suggests that the gamma rhythm depends not only on the suppressive normalization signal, but on the incoming excitatory drive as well, and could be a resonant phenomenon arising from the excitation–inhibition interaction [13],[18],[28],[29]. However, differences in the levels of excitation alone across stimulus conditions cannot explain these results, because changes in excitation modulate power in a broad frequency band including the high-gamma band (see Discussion for more details).

**Figure 6:**
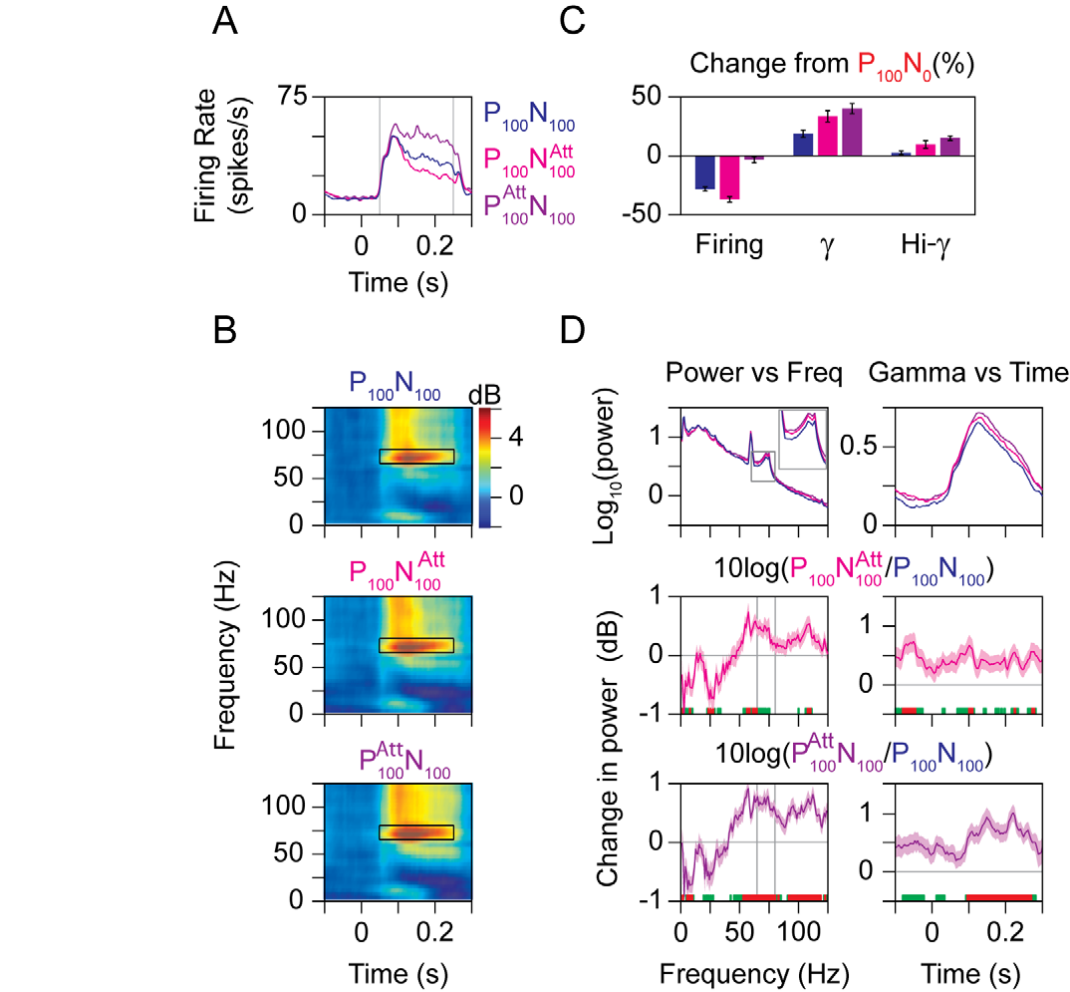
Attention increases gamma power. (A) Average firing rates of 96 neurons when a preferred and null stimulus was presented at 100% contrast inside the receptive field, while the monkeys attended to a stimulus outside the receptive field moving at an intermediate direction (P_100_N_100_), the null stimulus inside the receptive field (P_100_N_100_^Att^), or the preferred stimulus (P_100_^Att^N_100_) inside the receptive field. (B) Time-frequency power difference spectra for the three conditions described in (A). (C) Percent change in firing rates (Firing), gamma power (γ), and high-gamma power (Hi-γ) relative to the P_100_N_0_ condition, for the three attentional conditions described in (A). (D) LFP power as a function of frequency (left column) and gamma power as a function of time (right column). The top plot shows the raw power for different attention conditions described in (A). The middle plot shows the comparison between P_100_N_100_^Att^ versus P_100_N_100_, while the bottom plot shows the comparison between P_100_^Att^N_100_ versus P_100_N_100_. Stimuli for these plots are identical, so the difference is purely due to attention. Same convention as in Figure 4B and 4D.

Next, we studied the effect of shifting the focus of attention under identical stimulus conditions (Figure 1B, “Spatial Attention Protocol”). Figure 6A shows the average firing rates of the 96 neurons when two stimuli at 100% contrast moving in the preferred and null directions were presented inside the receptive field, while the animal focused on a stimulus outside the receptive field (P_100_N_100_; dark blue trace) or on the null (P_100_N_100_^Att^; magenta) or preferred (P_100_^Att^N_100_; violet) stimulus inside the receptive field. This attentional manipulation allowed us to dissociate the dependence of gamma power on normalization versus firing rate modulations. This is because the response of the neuron shifted toward the response elicited when the attended stimulus was presented alone, and therefore decreased when attending to null (P_100_N_100_^Att^) and increased when attending to preferred (P_100_^Att^N_100_) compared to the P_100_N_100_ condition [30],[31]. In contrast, the strength of normalization increased for both P_100_N_100_^Att^ and P_100_^Att^N_100_ conditions (compared to the P_100_N_100_ condition) because attention was directed to a stimulus inside the receptive field instead of outside. This was indeed reflected in the gamma power, whose strength increased when attention was directed inside the receptive field for both the P_100_N_100_^Att^ and P_100_^Att^N_100_ conditions (Figure 6B; compare first versus second/third row). Figure 6C shows the normalized firing rate (Firing), gamma power (γ), and high-gamma power (Hi-γ) for the P_100_N_100_, P_100_N_100_^Att^, and P_100_^Att^N_100_ conditions (normalized with respect to P_100_N_0_ as before). The firing rate decreased by 28.6%±1.8% (dark blue bar) when a null stimulus was added to the receptive field and decreased by 37.1%±2.3% when attention was directed to that null stimulus (magenta bar). Attention to the preferred stimulus largely counteracted the presence of the null stimulus, leaving a decrease of only 3.3%±2.6% from the preferred only stimulus (violet bar). On the other hand, gamma power increased by 18.8%±3.1% when the null stimulus was added, 33.6%±4.8% when this null stimulus was attended, and 40.1%±4.3% when the preferred stimulus was attended (all changes compared to the P_100_N_0_ condition). The increase of 12.9% in the gamma power from P_100_N_100_ to P_100_N_100_^Att^ was highly significant (*p* = 3.5×10^−5^, *N* = 96, *t* test). When analyzed separately, the increase was 9.0% (*p* = 0.0017, *N* = 55, *t* test) for Monkey 1 and 18.2% (*p* = 0.005, *N* = 41, *t* test) for Monkey 2. The increase from P_100_N_100_^Att^ to P_100_^Att^N_100_ was 8.1% for the pooled data (*p* = 0.02, *N* = 96, *t* test), 4.4% for Monkey 1 (*p* = 0.35, *N* = 55, *t* test), and 13.3% for Monkey 2 (*p* = 0.04, *N* = 41, *t* test). Thus, manipulations of attention that increased normalization increased gamma power even when they decreased the firing rate, suggesting that the effects of attention on gamma power may be an indirect consequence of its direct effect on normalization.

Unlike manipulations of normalization, manipulations of attention changed the power at non-gamma frequencies also. For example, power in the high-gamma range increased by 2.5%±1.5% when the null stimulus was added, 9.6%±3.1% when this null stimulus was attended, and 15.0%±1.8% when the preferred stimulus was attended (Figure 6C, “Hi-γ”). The increases of 6.9% from P_100_N_100_ to P_100_N_100_^Att^ and 4.9% from P_100_N_100_^Att^ to P_100_^Att^N_100_ were both significant (*p* = 0.03 and *p* = 0.02, *N* = 96, *t* test).

To study the effect of attention at different frequencies in more detail, we plotted the power between 50 and 250 ms as a function of frequency (Figure 6D; left column) and the gamma power as a function of time (Figure 6D, right column) for different attention conditions. The top row shows the raw power, while the middle and bottom rows show the change in power for the P_100_N_100_^Att^ versus P_100_N_100_ condition and P_100_^Att^N_100_ versus P_100_N_100_ conditions, respectively. Attention increased the power in a broad frequency band above 50 Hz and decreased power below 30 Hz (left column, middle and bottom rows). As a function of time, gamma power was elevated throughout the duration of the trial irrespective of stimulus onset for the P_100_N_100_^Att^ versus P_100_N_100_ condition (middle row, right column), but showed a larger increase after stimulus onset for the P_100_^Att^N_100_ versus P_100_N_100_ condition (bottom row, right column). Results obtained from multitaper analysis were very similar (not shown).

**Figure 7:**
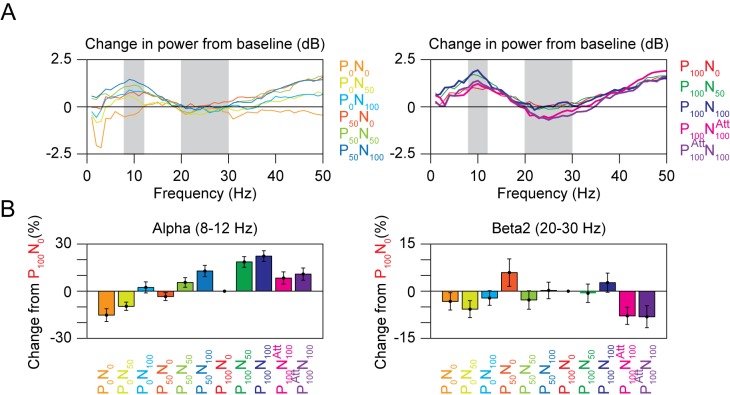
Effect of normalization and attention at low frequencies. (A) Change in power from baseline, for nine normalization and two attention conditions. The data are shown in two plots for clarity. Alpha and beta2 bands are shaded in gray. (B) Change in power from the P_100_N_0_ condition in the alpha (left) and beta2 (right) band.

We observed a pronounced suppression at low frequencies (<30 Hz) with attention, as shown in Figure 6B and 6D. To study the effects of normalization and attention at low frequencies, we plotted the change in power from baseline for different normalization and attention conditions (Figure 7A). From the time-frequency difference plots (Figures 3B and 6B), two prominent features were observed at low frequencies. First, we observed an increase in power at ∼10 Hz at ∼100 ms, probably reflecting the stimulus-induced transient. Second, we observed a pronounced suppression in power between 20 and 30 Hz. Figure 7B shows the change in power (from the P_100_N_0_ condition as before) in the alpha (8–12 Hz; left panel) and beta2 (20–30 Hz; right) bands for different normalization and attention conditions. For the Normalization conditions (from P_0_N_0_ through P_100_N_100_), alpha power increased with the strength of normalization, probably because the stimulus-induced transient reflected the overall population activity that increased with increasing normalization (Figure 3B). The beta2 band did not show any significant modulation with normalization (Figure 7B, right panel). This can also be seen in Figure 3B, where the blue patches reflecting the beta2 decrease have approximately the same intensity. Even though this patch appears missing in the P_0_N_0_ condition, it is only because power at other frequencies changes by a similar proportion—that is, other frequencies also have a similar shade of blue, so the color contrast is not salient (compare the orange trace in Figure 7A that has no dip in the beta2 range with other traces that show a prominent dip). On the other hand, attention decreased the power in both alpha and beta2 ranges (Figures 6B and 7), consistent with a large number of prior studies [5],[12],[32],[33].

Finally, we studied whether the increase in gamma power due to attention can be explained through normalization on a neuron-by-neuron basis. Neurons in area MT have a variable change in firing rate when a null stimulus is added to a preferred stimulus in their receptive field—for some neurons, the firing rate decreases substantially, while for others there is hardly any decrease, which can be explained by the variability in the strength of the normalization (the tuned normalization model is summarized in Text S1) [24]. The strength of normalization can be approximated as α = (firing rate(P_100_N_0_)/firing rate(P_100_N_100_))−1 (Text S1). Previous studies have shown that α is strongly correlated with the overall attentional modulation in firing rates [measured as (P_100_^Att^N_100_−P_100_N_100_^Att)^/(P_100_^Att^N_100_+P_100_N_100_^Att^)] [19],[24]. We therefore studied whether α can also predict the attentional modulation in gamma power.

**Figure 8:**
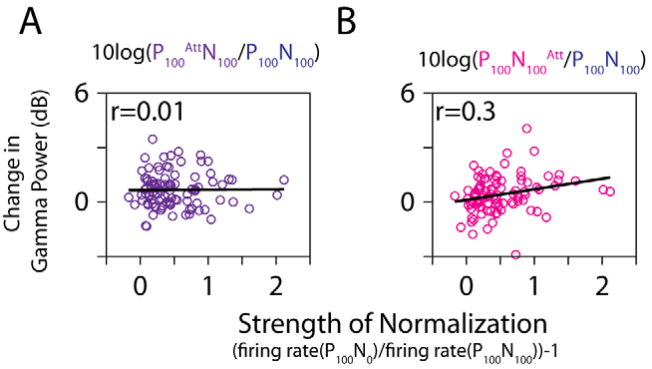
Comparison of normalization and attention on a neuron-to-neuron basis. (A) For each neuron, the degree of normalization (α), defined as firing rate(P_100_N_0_)/firing rate (P_100_N_100_)−1 (*x*-axis), is plotted against the relative change in gamma power when attention is directed to a preferred stimulus versus outside: log10(gamma power(P_100_^Att^N_100_)/gamma power(P_100_N_100_)) (*y*-axis). The Spearman rank correlation is indicated on the top. The black line indicates the best fit obtained through linear regression. (B) Same analysis when attention is directed to the null stimulus.

Figure 8A plots the relationship between the increase in gamma power (measured in dB) when attention was directed to the preferred stimulus versus outside (P_100_^Att^N_100_ versus P_100_N_100_), as a function of the normalization strength (α). Neurons demonstrating a stronger normalization signal (α) should show a greater attentional modulation in gamma power. However, these two parameters were not correlated (ρ = 0.01, *p* = 0.9, Spearman Rank test). This is because gamma power depends not only on the strength of normalization but also on the strength of the incoming excitation, and attention increases both these quantities. This issue can be partially resolved by studying the correlation between α and the increase in gamma power when attention was directed to the null stimulus (Figure 8B), because in this case attention increases the strength of normalization but does not substantially increase the strength of incoming excitation (because the null stimulus produces almost no response in neurons in area MT). In this case, the increase in gamma power was weakly but significantly correlated with α (ρ = 0.3, *p* = 0.003, *N* = 96, Spearman Rank test), although the correlation did not reach significance for Monkey 1 when the analysis was done separately for each monkey (Monkey 1: ρ = 0.21, *p* = 0.13, *N* = 55; Monkey 2: ρ = 0.37, *p* = 0.02, *N* = 41, Spearman Rank test). Thus, changes in firing rates from a pure manipulation of normalization (which were used to estimate α) were a weak but significant predictor of the changes in gamma power during a manipulation of attention, but only when attention modulated the normalization strength alone. Differences between the effects of normalization and attention on the power spectrum are addressed in more detail in the Discussion.

## Discussion

This study integrates a number of other results to directly link normalization strength and gamma power and provides an alternate explanation for the increase in gamma typically observed in higher cortical areas due to attention. Prior studies have shown that gamma power is modulated by incoming excitation and inhibition and could be a resonant phenomenon arising from their interaction [13],[16],[18],[28],[29],[34]. Some models of normalization are based on such excitation–inhibition interactions [21],[22], although other models of normalization may operate without inhibition, as described below. Finally, previous studies have shown that effects of attention and normalization on a particular neuron are tightly correlated [18],[28], suggesting that attention could change the strengths of excitation and normalization [19],[20]. The present study integrates these results—we first link gamma power to normalization strength while keeping attention constant, and then use an attention paradigm to show that the increase in gamma power due to attention could be explained at least in part by the effect of attention on normalization strength.

### Comparison with Other Models of Attention

Early models of attention such as the biased competition model [35]–[37] suggested that when multiple stimuli are presented inside the receptive field of a neuron, they activate different neural assemblies that compete for high-level representation, and attention biases the competition in favor of the attended stimulus. These models, however, fail to explain the effect of attention on neural responses when a single stimulus is present inside the receptive field [38]. Other types of models such as the flexible input gain model [23],[39] operate by changing the relative weights of inputs into a neuron, without changing the rules by which these inputs are integrated together. The input gain model can explain the increase in firing rates observed when a single stimulus is presented, as well as the competitive behavior when multiple stimuli are presented [23],[39]. In this model, the response of a neuron when a preferred and a null stimulus are both presented is given by *R_P,N_* = λ((β*_P_R_P_*)*^n^*+(β*_N_R_N_*)*^n^*)^1/*n*^, where *R_P_* and *R_N_* are the responses when the preferred and null stimuli are presented alone, β*_P_* and β*_N_* are the attentional gains applied to each input, *n* incorporates nonlinear summation (*n* = 1 for linear; *n* = infinity for winner-take-all), while λ is a scaling term. However, input gain or biased competition models cannot easily explain the decrease in firing rates when a null stimulus is attended if the null stimulus produces no response to begin with, which was the case in our dataset (Figure 3A, left panel). Specifically, if *R_N_* = 0, the input gain model reduces to *R_P,N_* = λβ*_P_R_P_*, which cannot explain the decrease in firing rate observed when attention is directed to the null stimulus unless the scaling parameter λ changes with the direction of attention (preferred versus null). The normalization model of attention (Text S1) also acts by multiplying the inputs by a gain term and, in this regard, is similar to the input gain model. In addition, the responses are divided (normalized) by a term that depends on the null stimulus contrast and null attentional gain, even if the null stimulus produces no response. The normalization model can effectively change the scaling term of the gain model (λ) with changing attention, and therefore can explain a wider range of experimental results [19],[20],[24].

### Broadband Versus Band-Limited Gamma

Several studies have shown that increasing the strength of incoming excitation increases the power in a broad frequency band above ∼30 Hz, including the gamma and high-gamma band, and this broad-band increase in power is correlated with the firing rate of the neural population near the microelectrode [40],[41]. This is different from “band-limited” gamma rhythm that is often visible in the power spectrum as a distinct “bump” with a bandwidth of ∼20 Hz, which is sustained by a inhibitory network [28],[42],[43], and may not be correlated with spiking activity [14],[34],[41]. Our results show that normalization increases band-limited gamma, while attention increases both excitation and normalization and therefore affects the power over a broader frequency range.

Band-limited gamma may not always be observed during an attention task. For example, Khayat and colleagues [12] recorded from area MT of monkeys engaged in an attention task while presenting two random dot patterns—one moving in the null direction at 100% contrast paired with another moving in the preferred direction at varying contrasts, thus changing both excitation and normalization across stimulus conditions. The authors observed a broadband change in power in the gamma and high-gamma range, but no band-limited gamma. A similar spectral profile was observed in another recording from area MT where random dot patterns were used [17]. Indeed, most early studies that showed a salient band-limited gamma used one of two types of stimuli—gratings or oriented bars [44]–[46]. Most studies showing an effect of attention on band-limited gamma have also used either gratings or bars [1],[4],[5],[32],[47]. The absence of a prominent band-limited gamma rhythm in a demanding attention task [12] suggests that band-limited gamma may not play a functional role in attention and may not even be a fundamental marker of normalization or excitatory–inhibitory interactions. Instead, it could be a rhythm that is generated under special stimulus conditions and may reflect excitatory–inhibitory interactions within those restricted conditions.

### Gamma Modulation from Different Types of Normalization

In this paper we have only considered a specific type of normalization, which is due to the addition of a nonoverlapping null stimulus inside the receptive field. Response suppression also occurs when an overlapping null stimulus is added to a preferred stimulus inside the receptive field, or when the stimulus size exceeds the classical receptive field (surround suppression). Whether these forms of suppression involve the same normalization circuit is unclear. For example, although earlier models of suppression produced by overlapping orthogonal gratings were based on inhibition [21],[22], recent models have explained this suppression without inhibition (for a review, see [48]). Consistent with this, a recent paper has shown that superimposing a null grating on a preferred grating *decreases* the gamma power in the primary visual cortex (V1), and surprisingly, also increases the gamma center frequency [49]. It is possible that superimposed and nonoverlapping orthogonal gratings produce suppression by different mechanisms, with only the latter requiring inhibition.

Similarly, the presentation of a stimulus that is larger than the classical receptive field suppresses the response, although this manipulation increases the gamma power and decreases the gamma oscillation frequency in V1 [16]. The mechanism of surround suppression is unclear, with some studies showing an increase in incoming excitation and inhibition [50] and others showing the opposite effect [51]. Similarly, the cortical sites where normalization acts are also unclear. Earlier models assumed that normalization occurred simultaneously in multiple areas (V1 and MT; [52],[53]). However, properties of some types of opponent motion suppression differ between V1 and MT, which has been explained by a mechanism in which suppression arises in area MT [54]. On the other hand, responses of MT neurons that respond to the global motion of plaids (but not to the constituent component motion) were explained by a model where divisive normalization instead occurred in V1 [55]. Chalk and colleagues [5] have recently shown that gamma power *decreases* in area V1 with increasing attention, although under identical conditions gamma increases in V4. The differences could be due to the ways normalization is implemented in different cortical areas (see [5] for a more detailed discussion).

In summary, the normalization signal that is involved in response suppression could be computed using different mechanisms, depending on the specific stimulus properties and cortical area. At present, it is unclear how universal the relationship between gamma and normalization described in this article is; that is, whether other forms of normalization would also modulate gamma power in a similar way. Similarly, although the stimulus configuration used in this article (nonoverlapping orthogonal stimuli inside the receptive field) is a common design used in several attention studies [24],[30],[31],[35],[37], the relationship between attention and gamma when other forms of normalization may be operating remains an open question.

### Effects of Normalization and Attention on the Power Spectrum

In our data, manipulations of normalization strength affected only the gamma range (and very low frequencies that likely reflected a stimulus transient). Attention, on the other hand, decreased power at low frequencies, consistent with prior studies [12],[32],[33] and increased power in the gamma and high-gamma ranges. As described above, a broadband increase in gamma and high-gamma power is correlated with the firing rate of the neural population near the microelectrode [40],[41]. However, in this study we observed an increase in gamma and high-gamma power even when attention was directed to the null stimulus. This is at odds with a previous study where gamma and high-gamma power decreased, consistent with the decrease in firing rate [12]. There are several factors that may have contributed to this difference. First, Khayat and colleagues [12] measured gamma power 510–1,010 ms after stimulus onset, while we measured gamma power between 50 and 250 ms after stimulus onset. It is possible that stimulus onset excites the entire population transiently, before suppressive and attention-related mechanisms take over to modify the responses of the neural population. The effect would be a transient increase in overall firing followed by a reduction in firing of the population, which may explain why high-gamma power is high initially (when we recorded) but lower in the steady state (when Khayat and colleagues recorded). Another factor may be the spatial spread of attention. As described earlier, high-gamma power depends on the firing rate of the overall population near the microelectrode, not just of the neuron being recorded from the microelectrode. Directing attention to the null stimulus inside the receptive field has two opposing effects: an increase in the firing rate of most neurons in the attended cortical region, and a reduction in the firing rate of neurons whose receptive fields contained both the preferred and null stimuli (such as the neurons shown in Figure 6A). Depending on the focus of attention, the overall population activity could either increase or decrease. Importantly, the changes in high-gamma power with attention do not influence the main result of this article, which is the increase in band-limited gamma power with increasing normalization strength. Because the stimuli used by Khayat and colleagues did not produce a salient band-limited gamma rhythm (see above), the results between the two studies cannot be compared directly.

The lack of change in high-gamma power with increasing normalization strength (Figures 3B and 5B) can be explained similarly. A single stimulus activates a population of neurons, whose firing rate decreases when a second orthogonal stimulus is added (due to normalization and surround suppression). However, the second stimulus also activates another population of neurons. The overall population firing recorded by the microelectrode depends on the stimulus size, the size of the receptive field, suppressive surround and normalization pool, as well as the cortical spread of the population activity that is picked up by the microelectrode. It is possible that the overall population firing rate did not change appreciably when a second stimulus was added in our normalization protocol, so that high-gamma power did not change.

The gamma peak was observed between 65 and 80 Hz, a frequency range that is slightly above the traditional gamma range (30–60 Hz) and that overlaps with the high-gamma band [41],[56]. This could be due to the early time window for analysis (because the stimulus presentation was for a short duration), because gamma peak frequency is higher after stimulus onset and decreases with time (for example, see Figure 1H of [41]). This is also consistent with a previous report that showed gamma oscillations at ∼50 Hz when analysis was done at a late interval (>300 ms) but a peak at 65 Hz when analysis was done at an early period ([1], compare their Figure 1 versus 4). In addition, gamma center frequency varies from subject to subject depending on the resting GABA concentration [57], and also depends on stimulus parameters such as size [16],[34] and contrast [13]. Although the center frequency of the gamma rhythm was relatively high, it could be dissociated from high-gamma activity (related to population firing) based on the spectral profile because gamma rhythm between 65 and 80 Hz had a distinct bump in the power spectrum while the high-gamma activity had a broadband profile with no distinct peak. Nonetheless, because the effect of spiking activity is detectable above ∼50 Hz in the LFP and becomes progressively more significant with increasing frequency [41], the increase in gamma power due to attention could partly be due to the increase in the population firing rate. In addition, as discussed above, gamma power depends not only on suppressive normalization, but also on the strength of the incoming excitation, and its precise relation with excitation and inhibition is unknown. Consequently, the increases in gamma power due to attention and to normalization were not tightly correlated in our data (unlike the tight correlation observed in firing rates as described in [19],[24]). Only when attention was directed to the null stimulus, for which the increase in the incoming excitation was less (although not zero, because the high-gamma power increased significantly), could we observe a weak correlation between attention and normalization (Figure 8B).

In summary, our study shows that changes in the strength of normalization, which occur during attentional modulation, can also change the gamma power, although the precise nature of the relationship between normalization and gamma remains to be established. Changes in gamma power in an attention task due to changes in the underlying normalization strength must be accounted for before a more advanced functional role for gamma in the formation of communication channels [3],[10] or binding of stimulus features [7],[8] can be unequivocally established.

## Materials and Methods

### Ethics Statement

All procedures related to animal subjects were approved by the Institutional Animal Care and Use Committee of Harvard Medical School.

### Animal Preparation and Behavioral Task

This study uses the same dataset as used by Ni and colleagues [24]. Data were collected from two male rhesus monkeys (*Macaca mulatta*) that weighed 8 and 12 kg. A scleral search coil and a head post were implanted under general anesthesia. After recovery, each animal was trained to do an orientation change detection task. The animal was required to hold its gaze within 1.0° from the center of a small fixation target while a series of drifting Gabor stimuli were flashed at three locations: two within the receptive field of the MT neuron being recorded and one at a symmetric location on the opposite side of the fixation point from the receptive field. All three Gabors were centered at the same eccentricity from the fixation point, and the Gabors were identical except for their contrast and drift direction. The two stimulus locations in the receptive field were separated by at least 5 times the SD of the Gabors (mean Gabor SD, 0.45°; SD of Gabor SD, 0.04°; range, 0.42–0.50°; mean separation of Gabor centers, 4.2°; SD, 0.86°; range, 2.2–6.9°). The stimuli were presented on a gray background (42 cd/m^2^), which had the same mean luminance with the Gabors, on a gamma-corrected video monitor (1024×768 pixels, 75 Hz refresh rate).

The animal was cued to attend to one of the three locations in blocks of trials and to respond when a Gabor with a different orientation appeared there (the target), ignoring any orientation changes at uncued locations (distractors), which occurred with the same probability as changes at the cued location. The animal indicated its response by making a saccade directly to the target location within 100–600 ms of its appearance. Correct responses were rewarded with a drop of juice or water. The target location was cued by a yellow annulus at the beginning of each trial as well as by instruction trials. Instruction trials consisted of a series of Gabor stimuli that appeared in only one location. Two instruction trials were inserted each time the cued location changed.

Gabors were presented synchronously in all three locations for 200 ms, with successive stimuli separated by periods with pseudorandom durations of 158–293 ms. During each presentation, one Gabor inside the receptive field moved in the preferred direction of the neuron, while the other Gabor inside the receptive field moved in the opposite (null) direction. The Gabor outside the receptive field moved in an orthogonal (intermediate) direction. The “Normalization” and “Spatial Attention” protocols differed in the location of the cue (outside versus inside the receptive field) and the number of contrasts used for each stimulus (three versus two). For the Normalization protocol (Figure 1A), the monkey attended to the stimulus outside the receptive field, and all Gabors could take one of three contrast values: 0%, 50%, or 100% (the target stimulus had either 50% or 100% contrast). This created nine different stimulus conditions inside the receptive field, as shown in Figure 3 (for each condition, we pooled data for the three different contrast levels for the Gabor outside the receptive field). For the Spatial Attention protocol (Figure 1B), the monkey attended to one of the locations inside the receptive field (which could have either the preferred or null stimulus in different presentations). All Gabors had either 0% or 100% contrast (target stimulus always had 100% contrast). We only used the stimulus condition for which both the preferred and null stimuli inside the receptive field had 100% contrast because that configuration showed the largest effect of attention.

The stimulus at a given location inside the receptive field could either be the preferred or null stimulus across presentations within the same trial (Figure 1). For a subset of data recorded from Monkey 1 (45 out of 68 neurons), the stimulus direction was fixed for a given location, so that the preferred stimulus always appeared in the bottom half of the receptive field while the null stimulus always appeared on top. The results shown in the article were similar for this modified version of the task; the data were pooled.

The timing of the target appearance in each trial was selected from an exponential distribution (flat hazard function for orientation change) to encourage the animal to maintain constant vigilance throughout each trial. However, trials were truncated at 6 s if the target had not appeared (∼20% of trials), in which case the animal was rewarded for maintaining fixation up to that time. The orientation change was adjusted for each stimulus configuration using an adaptive staircase procedure (QUEST; [58]) to maintain a behavioral performance of 82% correct [hits/(hits+misses); range, 57%–93%] across all target locations [the average orientation change for targets and distractors were 50±12° and 52±7° for Monkeys 1 and 2 (mean±SD)]. Both monkeys had fast reaction times (245±13 and 195±7 ms; mean ± SD), which, coupled with the large attentional modulation observed in the firing rates, suggested that they were paying close attention to the stimuli.

### Data Collection

Recordings were made using glass-insulated Pt-Ir microelectrodes (∼1 MΩ at 1 kHz) in area MT (axis ∼22–40° from horizontal in a parasagittal plane). A guide tube and grid system [59] was used to penetrate the dura. Spikes and LFP were recorded simultaneously using a Multichannel Acquisition Processor system by Plexon Inc. with a head-stage with gain 20 (Plexon Inc. HST/8o50-G20). Signals were filtered between 250 Hz and 8 kHz, amplified and digitized at 40 kHz to obtain spike data. For the LFP, the signals were filtered between 0.7 and 170 Hz, amplified and digitized at 1 kHz. We used the FPAlign utility program provided by Plexon Inc. to correct for the filter induced time delays (http://www.plexon.com/downloads). The headstage HST/8o50-G20 has low input impedance, which can lead to a voltage divider effect at low frequencies (Figure 2B shows this effect at frequencies below ∼5 Hz) [60]. This is unlikely to affect our results because this effect is much less prominent in the frequency range of interest (65–80 Hz) and we always compared data across different stimulus conditions that had the same filter settings.

Once a single unit was isolated, the receptive field location was estimated using a hand-controlled visual stimulus. Computer-controlled presentations of Gabor stimuli were used to measure tuning for direction (eight directions) and temporal frequency (five frequencies) while the animal performed a fixation task. The temporal frequency that produced the strongest response was used for all of the Gabors. The temporal frequency was rounded to a value that produced an integral number of cycles of drift during each stimulus presentation, so that the Gabors started and ended with odd spatial symmetry, such that the spatiotemporal integral of the luminance of each stimulus was the same as the background. Spatial frequency was set to one cycle per degree for all of the Gabors. The preferred Gabor was used to quantitatively map the receptive field (three eccentricities and five polar angles) while the animal performed a fixation task. The two stimulus locations within the receptive field were chosen to be at equal eccentricities from the fixation point and to give approximately equal responses, and the third location was 180° from the center point between the two receptive field locations, at an equal eccentricity from the fixation point as the other locations.

Cells were included in the analysis if they were held for at least nine repetitions (mean 41 repetitions) of each stimulus/attention combination used in this article. The response for each condition was taken as the average rate of firing in a period 50–250 ms after stimulus onset. Target stimuli and stimuli presented with a distractor were excluded from analysis, as were stimuli that appeared after the target. Additionally, the first stimulus presentation in each trial was excluded from analysis to reduce variance arising from stronger responses to the start of a stimulus series. Instruction trials were excluded from data analysis.

Spikes and LFP were collected from 68 sites from Monkey 1 and 50 from Monkey 2. Out of these, 13 and 9 sites were discarded because either the LFP signal was too large and saturated frequently or was too weak (<10 µV). The results were similar (and individually significant) for the two monkeys, and the gamma oscillations were also in the same frequency range; the data were pooled.

### Data Analysis

Time-frequency analysis was performed using the Matching Pursuit algorithm [61]. Due to the rapid presentation of the stimuli (duration of 200 ms with interstimulus interval of 158–293 ms), the LFP signal had transient activity associated with stimulus onset/offset. This required time-frequency analysis over short intervals (i.e., good temporal but poor spectral resolution). On the other hand, line noise at 60 Hz and the monitor refresh rate at 75 Hz produced signals at constant frequency (60 and 75 Hz), which were sustained for long periods (Figure 2). To represent such signals, time-frequency analysis should be done over long intervals (to achieve good spectral resolution at an expense of temporal resolution). These requirements are difficult to fulfill using traditional signal processing techniques such as short-time Fourier Transform or multi-tapering, but can be addressed using multiscale analysis techniques such as Matching Pursuit [61]. In this method, we start with an overcomplete dictionary of Gabor functions that have a wide range of time-frequency resolutions, including delta functions and sinusoids. The functions that best represent the signal are chosen for representation using an iterative procedure [26]. In this article, Matching Pursuit analysis was done on 1-s-long LFP segments, so the line noise at 60 Hz and the weaker noise at the monitor refresh rate of 75 Hz were captured by sinusoidal functions, which had a spectral resolution of ∼1 Hz, resulting in sharp lines at 60 and 75 Hz (Figure 2). Although Matching Pursuit algorithm provides better resolution to resolve transient and sustained activity, the results obtained using the multitaper method were similar (Figure S1).

### Construction of Figures

For each site, first a common “baseline power spectrum” was computed by averaging the power between 100 to 0 ms before stimulus onset for all nine normalization conditions (denoted by Baseline(ω); Figure 2B, black line). For Figure 3B and 6B, the time-frequency power spectra were normalized by this baseline power [10.(log(Power(t,ω)−log(Baseline(ω))]. Note that all the plots were normalized by the same baseline power (average of the baseline power obtained from the nine normalization conditions), which eliminates the possible effects of differences in baseline power across conditions. We showed changes in LFP power instead of raw power because LFP has a prominent “1/f” structure with more energy at low frequencies, which makes it difficult to observe any changes at higher frequencies in the raw time-frequency power spectra. Further, the difference spectra do not show the line and refresh-rate-related noise because this noise is present before stimulus onset also. The difference spectra were smoothed by averaging the power in every 4 time and frequency bins (essentially downsampling by a factor of 4 in both dimensions). This smoothing was done only for better visual display; all the power versus frequency/time plots (Figures 4, 5D, and 7A) as well as the power difference calculations (Figures 5, 6C, 7B, and 8) were done using raw data.

The gamma power was computed by summing the power between 65 and 80 Hz, but excluding the monitor refresh rate (between 74 and 76 Hz). Power from each condition was divided by the power for the P_100_N_0_ condition before averaging across neurons. High-gamma power was taken between 80 and 135 Hz because we observed a noise peak between 140 and 150 Hz, possibly arising from the stepper motor used to drive the microelectrodes when it was not moving, and the power above 150 Hz was attenuated by the low pass filter in the Plexon recording system.

## Supporting Information

Figure S1 Power spectra for different normalization and attention conditions, computed using the multitaper method. Comparable plots using Matching Pursuit (MP) are shown in Figures 2B and 6D. Baseline power is computed between 200 to 0 ms before stimulus onset to obtain the same frequency resolution as the remaining curves (as opposed to 100 to 0 ms for MP analysis), and therefore the baseline power is much greater than the P_0_N_0_ condition in this plot as compared to the results obtained using MP analysis (Figure 2B).
(TIF)

Text S1 Summary of the tuned normalization model with equations.
(DOC)

## Abbreviations

LFP: local field potential
MP: matching pursuit
MT: middle temporal
V1: primary visual cortex

## References

[1] Fries P, Reynolds JH, Rorie AE, Desimone R (2001) Modulation of oscillatory neuronal synchronization by selective visual attention. Science 291: 1560–1563. 11222864 10.1126/science.1055465

[2] Bichot NP, Rossi AF, Desimone R (2005) Parallel and serial neural mechanisms for visual search in macaque area V4. Science 308: 529–534. 15845848 10.1126/science.1109676

[3] Womelsdorf T, Schoffelen J-M, Oostenveld R, Singer W, Desimone R, et al (2007) Modulation of neuronal interactions through neuronal synchronization. Science 316: 1609–1612. 17569862 10.1126/science.1139597

[4] Gregoriou GG, Gotts SJ, Zhou H, Desimone R (2009) High-frequency, long-range coupling between prefrontal and visual cortex during attention. Science 324: 1207–1210. 19478185 10.1126/science.1171402 PMC2849291

[5] Chalk M, Herrero JL, Gieselmann MA, Delicato LS, Gotthardt S, et al (2010) Attention reduces stimulus-driven gamma frequency oscillations and spike field coherence in V1. Neuron 66: 114–125. 20399733 10.1016/j.neuron.2010.03.013 PMC2923752

[6] Pesaran B, Pezaris JS, Sahani M, Mitra PP, Andersen RA (2002) Temporal structure in neuronal activity during working memory in macaque parietal cortex. Nat Neurosci 5: 805–811. 12134152 10.1038/nn890

[7] Singer W (1999) Neuronal synchrony: a versatile code for the definition of relations? Neuron 24: 49–125, 49-65, 111-125. 10677026 10.1016/s0896-6273(00)80821-1

[8] Uhlhaas PJ, Pipa G, Lima B, Melloni L, Neuenschwander S, et al (2009) Neural synchrony in cortical networks: history, concept and current status. Front Integr Neurosci 3: 17. 19668703 10.3389/neuro.07.017.2009 PMC2723047

[9] Melloni L, Molina C, Pena M, Torres D, Singer W, et al (2007) Synchronization of neural activity across cortical areas correlates with conscious perception. J Neurosci 27: 2858–2865. 17360907 10.1523/JNEUROSCI.4623-06.2007 PMC6672558

[10] Fries P (2009) Neuronal gamma-band synchronization as a fundamental process in cortical computation. Annu Rev Neurosci 32: 209–224. 19400723 10.1146/annurev.neuro.051508.135603

[11] Henrie JA, Shapley R (2005) LFP power spectra in V1 cortex: the graded effect of stimulus contrast. J Neurophysiol 94: 479–490. 15703230 10.1152/jn.00919.2004

[12] Khayat PS, Niebergall R, Martinez-Trujillo JC (2010) Frequency-dependent attentional modulation of local field potential signals in macaque area MT. J Neurosci 30: 7037–7048. 20484646 10.1523/JNEUROSCI.0404-10.2010 PMC6632662

[13] Ray S, Maunsell JHR (2010) Differences in gamma frequencies across visual cortex restrict their possible use in computation. Neuron 67: 885–896. 20826318 10.1016/j.neuron.2010.08.004 PMC3001273

[14] Berens P, Keliris GA, Ecker AS, Logothetis NK, Tolias AS (2008) Comparing the feature selectivity of the gamma-band of the local field potential and the underlying spiking activity in primate visual cortex. Front Syst Neurosci 2: 2. 18958246 10.3389/neuro.06.002.2008 PMC2526275

[15] Jia X, Smith MA, Kohn A (2011) Stimulus selectivity and spatial coherence of gamma components of the local field potential. J Neurosci 31: 9390–9403. 21697389 10.1523/JNEUROSCI.0645-11.2011 PMC3133446

[16] Gieselmann MA, Thiele A (2008) Comparison of spatial integration and surround suppression characteristics in spiking activity and the local field potential in macaque V1. Eur J Neurosci 28: 447–459. 18702717 10.1111/j.1460-9568.2008.06358.x

[17] Liu J, Newsome WT (2006) Local field potential in cortical area MT: stimulus tuning and behavioral correlations. J Neurosci 26: 7779–7790. 16870724 10.1523/JNEUROSCI.5052-05.2006 PMC6674213

[18] Atallah BV, Scanziani M (2009) Instantaneous modulation of gamma oscillation frequency by balancing excitation with inhibition. Neuron 62: 566–577. 19477157 10.1016/j.neuron.2009.04.027 PMC2702525

[19] Lee J, Maunsell JHR (2009) A normalization model of attentional modulation of single unit responses. PLoS ONE 4: e4651 doi:10.1371/journal.pone.0004651. 19247494 10.1371/journal.pone.0004651 PMC2645695

[20] Reynolds JH, Heeger DJ (2009) The normalization model of attention. Neuron 61: 168–185. 19186161 10.1016/j.neuron.2009.01.002 PMC2752446

[21] Heeger DJ (1992) Normalization of cell responses in cat striate cortex. Vis Neurosci 9: 181–197. 1504027 10.1017/s0952523800009640

[22] Carandini M, Heeger DJ, Movshon JA (1997) Linearity and normalization in simple cells of the macaque primary visual cortex. J Neurosci 17: 8621–8644. 9334433 10.1523/JNEUROSCI.17-21-08621.1997 PMC6573724

[23] Ghose GM (2009) Attentional modulation of visual responses by flexible input gain. J Neurophysiol 101: 2089–2106. 19193776 10.1152/jn.90654.2008 PMC2695627

[24] Ni AM, Ray S, Maunsell JHR (2012) Tuned normalization explains the size of attention modulations. Neuron 73: 803–813. 22365552 10.1016/j.neuron.2012.01.006 PMC3292773

[25] Mitra PP, Pesaran B (1999) Analysis of dynamic brain imaging data. Biophys J 76: 691–708. 9929474 10.1016/S0006-3495(99)77236-X PMC1300074

[26] Jarvis MR, Mitra PP (2001) Sampling properties of the spectrum and coherency of sequences of action potentials. Neural Comput 13: 717–749. 11255566 10.1162/089976601300014312

[27] Sclar G, Maunsell JH, Lennie P (1990) Coding of image contrast in central visual pathways of the macaque monkey. Vision Res 30: 1–10. 2321355 10.1016/0042-6989(90)90123-3

[28] Whittington MA, Traub RD, Jefferys JG (1995) Synchronized oscillations in interneuron networks driven by metabotropic glutamate receptor activation. Nature 373: 612–615. 7854418 10.1038/373612a0

[29] Kang K, Shelley M, Henrie JA, Shapley R (2009) LFP spectral peaks in V1 cortex: network resonance and cortico-cortical feedback. J Comput Neurosci 10.1007/s10827-009-0190-2 PMC3050555 19862612

[30] Treue S, Maunsell JH (1996) Attentional modulation of visual motion processing in cortical areas MT and MST. Nature 382: 539–541. 8700227 10.1038/382539a0

[31] Lee J, Maunsell JHR (2010) Attentional modulation of MT neurons with single or multiple stimuli in their receptive fields. J Neurosci 30: 3058–3066. 20181602 10.1523/JNEUROSCI.3766-09.2010 PMC2850605

[32] Fries P, Womelsdorf T, Oostenveld R, Desimone R (2008) The effects of visual stimulation and selective visual attention on rhythmic neuronal synchronization in macaque area V4. J Neurosci 28: 4823–4835. 18448659 10.1523/JNEUROSCI.4499-07.2008 PMC3844818

[33] Foxe JJ, Snyder AC (2011) The role of alpha-band brain oscillations as a sensory suppression mechanism during selective attention. Front Psychol 2: 154. 21779269 10.3389/fpsyg.2011.00154 PMC3132683

[34] Jia X, Smith MA, Kohn A (2011) Stimulus selectivity and spatial coherence of gamma components of the local field potential. J Neurosci 31: 9390–9403. 21697389 10.1523/JNEUROSCI.0645-11.2011 PMC3133446

[35] Moran J, Desimone R (1985) Selective attention gates visual processing in the extrastriate cortex. Science 229: 782–784. 4023713 10.1126/science.4023713

[36] Desimone R, Duncan J (1995) Neural mechanisms of selective visual attention. Annu Rev Neurosci 18: 193–222. 7605061 10.1146/annurev.ne.18.030195.001205

[37] Reynolds JH, Chelazzi L, Desimone R (1999) Competitive mechanisms subserve attention in macaque areas V2 and V4. J Neurosci 19: 1736–1753. 10024360 10.1523/JNEUROSCI.19-05-01736.1999 PMC6782185

[38] McAdams CJ, Maunsell JH (1999) Effects of attention on orientation-tuning functions of single neurons in macaque cortical area V4. J Neurosci 19: 431–441. 9870971 10.1523/JNEUROSCI.19-01-00431.1999 PMC6782389

[39] Ghose GM, Maunsell JHR (2008) Spatial summation can explain the attentional modulation of neuronal responses to multiple stimuli in area V4. J Neurosci 28: 5115–5126. 18463265 10.1523/JNEUROSCI.0138-08.2008 PMC2720676

[40] Ray S, Crone NE, Niebur E, Franaszczuk PJ, Hsiao SS (2008) Neural correlates of high-gamma oscillations (60–200 Hz) in macaque local field potentials and their potential implications in electrocorticography. J Neurosci 28: 11526–11536. 18987189 10.1523/JNEUROSCI.2848-08.2008 PMC2715840

[41] Ray S, Maunsell JHR (2011) Different origins of gamma rhythm and high-gamma activity in macaque visual cortex. PLoS Biol 9: e1000610 doi:10.1371/journal.pbio.1000610. 21532743 10.1371/journal.pbio.1000610 PMC3075230

[42] Bartos M, Vida I, Jonas P (2007) Synaptic mechanisms of synchronized gamma oscillations in inhibitory interneuron networks. Nat Rev Neurosci 8: 45–56. 17180162 10.1038/nrn2044

[43] Cardin JA, Carlen M, Meletis K, Knoblich U, Zhang F, et al (2009) Driving fast-spiking cells induces gamma rhythm and controls sensory responses. Nature 459: 663–667. 19396156 10.1038/nature08002 PMC3655711

[44] Gray CM, König P, Engel AK, Singer W (1989) Oscillatory responses in cat visual cortex exhibit inter-columnar synchronization which reflects global stimulus properties. Nature 338: 334–337. 2922061 10.1038/338334a0

[45] Engel A, Konig P, Kreiter A, Singer W (1991) Interhemispheric synchronization of oscillatory neuronal responses in cat visual cortex. Science 252: 1177–1179. 2031188 10.1126/science.252.5009.1177

[46] Engel AK, Kreiter AK, König P, Singer W (1991) Synchronization of oscillatory neuronal responses between striate and extrastriate visual cortical areas of the cat. Proc Natl Acad Sci USA 88: 6048–6052. 2068083 10.1073/pnas.88.14.6048 PMC52019

[47] Bosman CA, Schoffelen J-M, Brunet N, Oostenveld R, Bastos AM, et al (2012) Attentional stimulus selection through selective synchronization between monkey visual areas. Neuron 75: 875–888. 22958827 10.1016/j.neuron.2012.06.037 PMC3457649

[48] Priebe NJ, Ferster D (2008) Inhibition, spike threshold, and stimulus selectivity in primary visual cortex. Neuron 57: 482–497. 18304479 10.1016/j.neuron.2008.02.005

[49] Lima B, Singer W, Chen NH, Neuenschwander S (2010) Synchronization dynamics in response to plaid stimuli in monkey V1. Cereb Cortex 20 7: 1556–73. 19812238 10.1093/cercor/bhp218 PMC2882822

[50] Haider B, Krause MR, Duque A, Yu Y, Touryan J, et al (2010) Synaptic and network mechanisms of sparse and reliable visual cortical activity during nonclassical receptive field stimulation. Neuron 65: 107–121. 20152117 10.1016/j.neuron.2009.12.005 PMC3110675

[51] Ozeki H, Finn IM, Schaffer ES, Miller KD, Ferster D (2009) Inhibitory stabilization of the cortical network underlies visual surround suppression. Neuron 62: 578–592. 19477158 10.1016/j.neuron.2009.03.028 PMC2691725

[52] Simoncelli EP, Heeger DJ (1998) A model of neuronal responses in visual area MT. Vision Res 38: 743–761. 9604103 10.1016/s0042-6989(97)00183-1

[53] Britten KH, Heuer HW (1999) Spatial summation in the receptive fields of MT neurons. J Neurosci 19: 5074–5084. 10366640 10.1523/JNEUROSCI.19-12-05074.1999 PMC6782635

[54] Qian N, Andersen RA (1994) Transparent motion perception as detection of unbalanced motion signals. II. Physiology. J Neurosci 14: 7367–7380. 7996182 10.1523/JNEUROSCI.14-12-07367.1994 PMC6576905

[55] Rust NC, Mante V, Simoncelli EP, Movshon JA (2006) How MT cells analyze the motion of visual patterns. Nat Neurosci 9: 1421–1431. 17041595 10.1038/nn1786

[56] Uhlhaas PJ, Pipa G, Neuenschwander S, Wibral M, Singer W (2011) A new look at gamma? High- (>60 Hz) γ-band activity in cortical networks: function, mechanisms and impairment. Prog Biophys Mol Biol 105: 14–28. 21034768 10.1016/j.pbiomolbio.2010.10.004

[57] Muthukumaraswamy SD, Edden RAE, Jones DK, Swettenham JB, Singh KD (2009) Resting GABA concentration predicts peak gamma frequency and fMRI amplitude in response to visual stimulation in humans. Proc Natl Acad Sci USA 106: 8356–8361. 19416820 10.1073/pnas.0900728106 PMC2688873

[58] Watson AB, Pelli DG (1983) QUEST: a Bayesian adaptive psychometric method. Percept Psychophys 33: 113–120. 6844102 10.3758/bf03202828

[59] Crist CF, Yamasaki DS, Komatsu H, Wurtz RH (1988) A grid system and a microsyringe for single cell recording. J Neurosci Methods 26: 117–122. 3146006 10.1016/0165-0270(88)90160-4

[60] Nelson MJ, Pouget P, Nilsen EA, Patten CD, Schall JD (2008) Review of signal distortion through metal microelectrode recording circuits and filters. J Neurosci Methods 169: 141–157. 18242715 10.1016/j.jneumeth.2007.12.010 PMC2292115

[61] Mallat SG, Zhang Z (1993) Matching pursuits with time-frequency dictionaries. IEEE Trans Signal Processing 41: 3397–3415.

