# Supplementary figures and images for: Strength of Gamma Rhythm Depends on Normalization
Source: PLoS Biol. 2013 Feb 5;11(2):e1001477. doi: 10.1371/journal.pbio.1001477 (PMC3564761; doi:10.1371/journal.pbio.1001477)

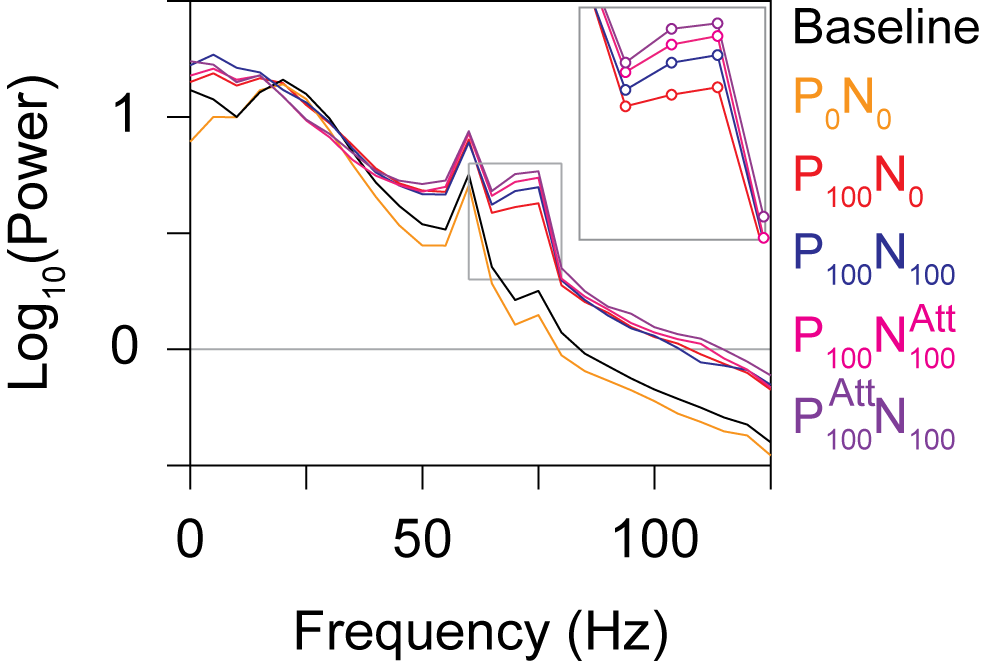

Supplement: Figure S1 — Power spectra for different normalization and attention conditions, computed using the multitaper method. Comparable plots using Matching Pursuit (MP) are shown in Figures 2B and 6D. Baseline power is computed between 200 to 0 ms before stimulus onset to obtain the same frequency resolution as the remaining curves (as opposed to 100 to 0 ms for MP analysis), and therefore the baseline power is much greater than the P0N0 condition in this plot as compared to the results obtained using MP analysis (Figure 2B). (TIF) [file pbio.1001477.s001.tif]
