# Supplementary material for: Strength of Gamma Rhythm Depends on Normalization
Source: PLoS Biol. 2013 Feb 5;11(2):e1001477. doi: 10.1371/journal.pbio.1001477 (PMC3564761; doi:10.1371/journal.pbio.1001477)
Supplement: Text S1 — Summary of the tuned normalization model with equations. (DOC) [file pbio.1001477.s002.doc]

Text S1

*Summary of the tuned normalization model*

Here we briefly describe the model that was used in a previous report to explain the changes in firing rates when normalization strength and attention were varied independently. In this model, the firing rate of a neuron is given by:

(1)

Here c_P_ and c_N_ are the contrasts of the preferred and null stimuli, L_P_ and L_N_ are the responses of the linear receptive field to the individual stimuli at unit contrast, and σ is a positive term that represents the semisaturation constant for the contrast response function of the neuron. Finally, α scales how much the null stimulus contributes to normalization relative to the preferred stimulus.

Note that if the normalization is untuned, a null stimulus contributes as much to the normalization pool as a prefered stimulus, so α=1. In this case, addition of a null stimulus at unit contrast to a preferred stimulus at unit contrast should decrease the response by a factor of ~2, especially for neurons with a small semi-saturation constant (σ<<1, which is the case for many MT neurons). This was not found to be the case. Instead, addition of a null stimulus had a large effect on the responses of some neurons, but others were hardly affected. This variability across neurons could be explained by variations in the tuned normalization parameter (α), because the response of a neuron would be hardly affected by the addition of a null stimulus if α=0, but would be reduced by a factor of 2 if α were close to 1.

In this model attention does not act by only changing the strength of normalization (denominator in equation 1), but instead by increasing the strength of incoming excitation (by a factor of β), which is also inherited by the normalization term:

(2A)

(2B)

Even though the attentional gain (β) does not depend on α, the overall effect of attention on the response of a neuron depends on α. In particular, attention has a small effect when α is small (essentially, if a neuron does not react to the presence of a null stimulus, it also does not react if this null stimulus is attended). The effects of normalization versus attention are therefore highly correlated across neurons, which can be explained by the variability in α across the neural population.

*Simplifications*

In our dataset, the neurons did not respond to the null stimulus (L_N_ ~0), and their responses saturated at low contrasts (σ<<1). Thus,

R_1,0_ ~ L_P_

R_1,1_ ~ L_P_/(1+α)

Solving, we get α ~ R_1,0_/R_1,1_ – 1 = (firing rate(P_100_N_0_)/firing rate(P_100_N_100_)) – 1.
